# Illustrating the effect of viscoelastic additives on cavitation and turbulence with X-ray imaging

**DOI:** 10.1038/s41598-018-32996-w

**Published:** 2018-10-08

**Authors:** I. K. Karathanassis, K. Trickett, P. Koukouvinis, J. Wang, R. Barbour, M. Gavaises

**Affiliations:** 1 School of Mathematics, Computer Science and Engineering, City, University of London, EC1V 0B London, UK; 2 Lubrizol Limited, Hazelwood, DE56 4AN Derby, UK; 3 Advanced Photon Source, Argonne National Laboratory, Lemont, IL 60439 USA

## Abstract

The effect of viscoelastic additives on the topology and dynamics of the two-phase flow arising within an axisymmetric orifice with a flow path constriction along its main axis has been investigated employing high-flux synchrotron radiation. X-ray Phase Contrast Imaging (XPCI) has been conducted to visualise the cavitating flow of different types of diesel fuel within the orifice. An additised blend containing Quaternary Ammonium Salt (QAS) additives with a concentration of 500 ppm has been comparatively examined against a pure (base) diesel compound. A high-flux, 12 keV X-ray beam has been utilised to obtain time resolved radiographs depicting the vapour extent within the orifice from two views (side and top) with reference to its main axis. Different test cases have been examined for both fuel types and for a range of flow conditions characterised by Reynolds number of 35500 and cavitation numbers (CN) lying in the range 3.0–7.7. It has been established that the behaviour of viscoelastic micelles in the regions of shear flow is not consistent depending on the cavitation regimes encountered. Namely, viscoelastic effects enhance vortical (string) cavitation, whereas hinder cloud cavitation. Furthermore, the use of additised fuel has been demonstrated to suppress the level of turbulence within the orifice.

## Introduction

Cavitation, i.e. liquid nucleation due to rapid flow depressurisation, is a flow phenomenon of violent and uncontrollable nature encountered in biological^1^ and industrial processes (see selectively^2–4^). Especially referring to industrial applications its onset is, on many occasions, accompanied by undesirable effects such as reduced device performance, deterioration of structural rigidity, vibrations and noise^5,6^. Therefore, elucidation of the cavitation topology and dynamics, as well as the underlying physical mechanisms, is of vital importance for the design of more effective flow devices and components.

Focusing in microfluidics devices, cavitation visualisation has been commonly achieved using optical techniques such as shadowgraphy or Schlieren imaging^7–10^. Yet, the small time- and length-scales, of the order of micrometers and microseconds at which the phenomenon develops pose practical challenges to its accurate resolution, since the vast majority of two-phase industrial flows is characterised by increased levels of turbulence with highly fluctuating features. In addition, the significant light scattering induced by even negligible, in terms of volume fraction, vapour quantities present, tend to obscure the obtained images and prevent full illustration of the, intertwining at some cases, nevertheless distinct forms of cavitation, such as sheet, cloud, occasionally with bubble shedding and vortical (string) cavitation^11,12^.

Visualisation of two-phase flows by taking into advantage the interaction of X-rays with matter is a novel technique employed in the last decade primarily the elucidation and quantification, in terms of vapour content, of external and spray-flows^13–15^, where the beam attenuation is low, compared to material-confined flows, and designated only by the density fluctuations of the liquid-vapour mixture. Few X-ray based studies exist in the literature examining wall-confined cavitating flows due to the technical difficulties associated with excessive beam attenuation due to its penetration to solid material. Bauer *et al*.^16^ quantified the average, yet 3-D, two-phase flow field inside an axisymmetric large-scale nozzle at cavitation numbers ranging from inception to super-cavitation was quantified through X-ray computed tomography (CT). More recently, time averaged X-ray measurements of the volume fraction distribution inside fuel-injector replicas have been obtained by Koukouvinis *et al*.^17^. The experimental investigations of Duke *et al*.^18,19^ are the only available in the literature offering time-resolved vapour faction measurements with regard to a cavitating nozzle flow. The experimental method in mention utilises an interpolation technique based on a matrix of point measurements, while synchrotron radiation and doping of the fuel employed with a contrast-enhancing agent were necessary to ensure adequate signal-to-noise ratio for the measurements.

The X-ray Phase Contrast Imaging (XPCI) method is based on the X-ray wave phase shift due to the Compton and Rayleigh scattering caused by the beam interaction with matter. A phase radiography is primarily designated by strong wave phase fluctuations, since the extent of scattering, i.e. the material refractive index, is orders of magnitude lower for X-rays compared to visible light in reference to hydrocarbon fuels^20^. The technique is capable of providing qualitative yet ultra high-speed data, which constitute suitable for highly fluctuating two-phase flows. Until now, it has been primarily employed to resolve spray flows produced by fuel injectors. The advantages of XPCI over optical methods in capturing the cavity topology and especially the dynamic evolution of interfacial perturbations has been demonstrated in reference to both wall-confined flows^21^ and spray flows^22^.

Viscoelastic surfactants have been widely utilised in applications related to the oil and gas industry, such as drilling and reservoir stimulation, heating and cooling applications, as well as household, e.g. detergents, and personal care products^23,24^. The dilution of polymers in concentrations of parts-per-million in the base liquid has been demonstrated to reduce the levels of turbulence and hence fluid drag in single-phase flows^25^. The underlying physical mechanism reflects on to the influence of the additional viscoelastic stress on the turbulence cascade sequence. In other words, the flexible additives dynamically stretch and rearrange their topology in the presence of flow shear, a behaviour macroscopically perceived as viscoelasticity^26^. The effect of viscoelastic additives has been found to be more profound in the boundary layer region, where turbulent friction losses have been found to decrease up to 80% compared to the respective of the base solvent for additives concentration of ppm^27^.

The effect of the rheological properties of viscoelastic fluids on the development and evolution of cavitation has not been thoroughly explored in the open literature. There are few bubble dynamics studies available focusing on the collapse of vapour bubbles in a viscoelastic, tissue-like medium^28–30^. It has been established that the bubble-collapse-induced liquid jet is of reduced momentum compared to a Newtonian liquid, while bubble oscillations are damped by viscosity and compressibility effects. With reference to applications at industrial scales, the study of Chahine *et al*.^31^ is the only one in the literature reporting that the injection of a polymer solution from a port located at the blade of a ship propeller has been found to delay the inception of tip vortex cavitation.

The interaction of turbulence, cavitation and rheological properties of viscoelastic fluids constitutes an open question to the fluid-mechanics community, as the presence of both vapour bubbles and viscoelastic micelles offer alternative routes of turbulent cascade to the dissipative scale. The present study is to the author’s knowledge the first to provide experimental data on the influence of Quaternary Ammonium Salt (QAS) additives on the turbulent two-phase flow emerging in an enlarged injector-replica orifice. Synchrotron radiation was utilised to provide time-resolved imaging of the highly transient cavitating structures for different cavitation regimes with exposure times of the order of nanoseconds, so as to capture the structures interfacial perturbations. The effect of additives on the flow turbulence level has also been quantified employing a technique similar to Particle Tracking Velocimetry (PTV)^32,33^ in order to estimate the local velocity fluctuations in the vapour region. The distinct difference between PTV and conventional Particle Image Velocimetry (PIV) is that seeding of the flow with tracking particles is not required by the former technique, since inherent flow features are tracked over time. PTV is therefore preferable in two-phase flows with phase-change, where the presence of seeding particles would possibly influence the cavitation inception and development, as also discussed in more detail in the Methods section and in^21^. Manipulation of cavitation dynamics using QAS additives would be of tremendous interest for various industrial sectors spanning from energy, automotive and marine to pharmaceutical and biomedical, as positive consequences of cavitation development could be exploited such as deposits de-agglomeration, spray atomization and focused ultrasound drug delivery^34^.

## Results

### QAS additives morphology

**Figure 1:**
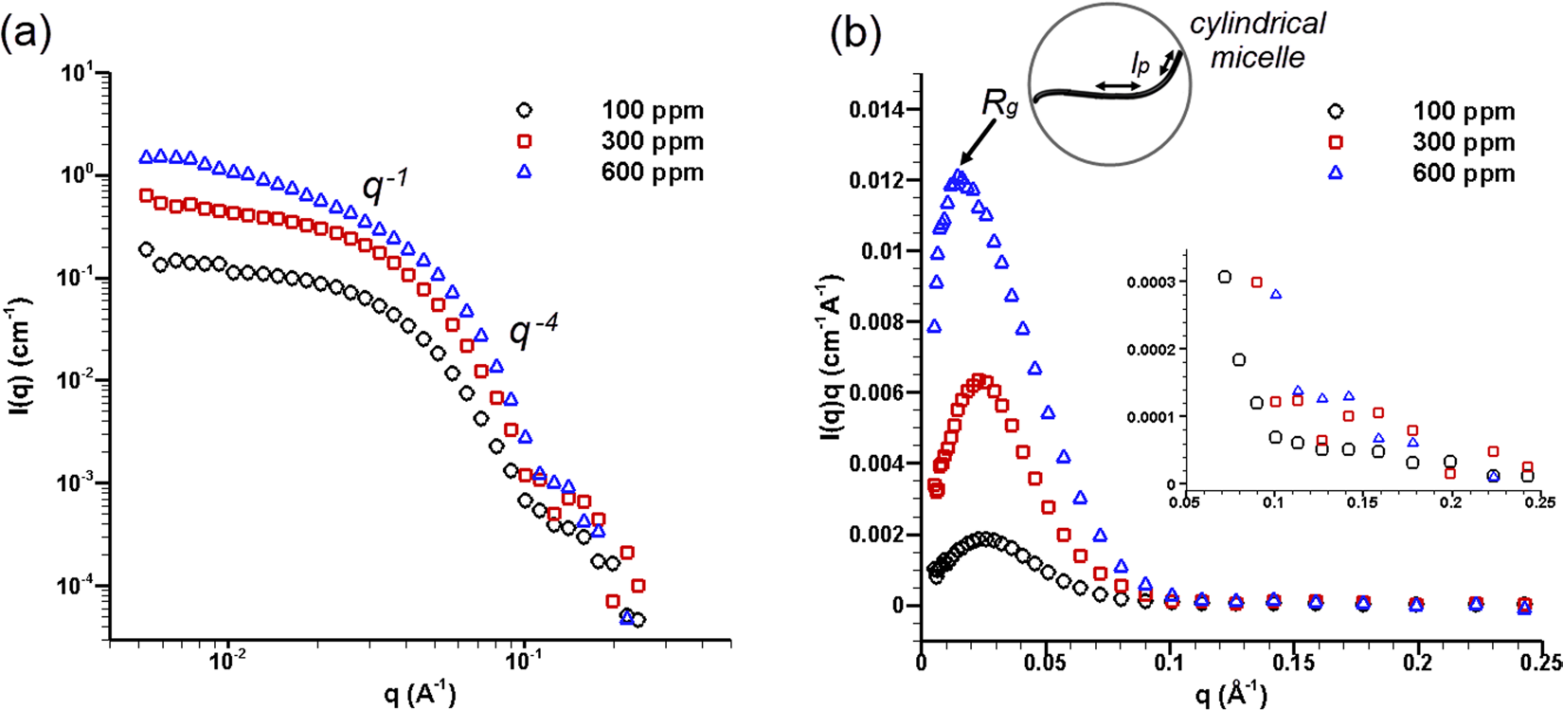
(**a**) SANS intensity variation with scattering vector q for different concentrations of QAS additives in the solvent. Different q regimes have been identified and the respective slopes are also annotated in the plot. (**b**) Holtzer plot, i.e. *I(q)q ~ q*, of the scattering data. The location of the characteristic maximum point is highlighted for each concentration. The inset corresponds to the micelle morphological features that can be extracted from the plot.

The viscoelastic behaviour of QAS additives, in essence constitutes an ensemble property designated by the topology and dynamic interactions of micellar structures forming in the nanoscale. Micelle morphology can be, in general, described by a number of characteristic length-scales, i.e. the gyration radius *R*_*g*_, overall length *L*, persistence length *l*_*p*_, i.e. the micelle part deemed as rigid, and cross-section radius *R*_*c*s_ (see inset of Fig. 1b)^23^.

Small Angle Neutron Scattering (SANS) has been performed in additised dodecane, a widely employed surrogate of diesel fuel, under stagnant conditions. Figure 1a depicts the intensity of the scattered neutrons *I* with the scattering vector defined as $q=4\pi /\lambda \,sin\,(\theta /2)$, where *λ* is the radiation wave and *θ* the scattering angle. The slope of the curve at different regions of q is indicative of the micellar structure. More specifically, at the region 0.01 < *q* < *0.03* a clear *q*^−1^ slope exists for the 600 ppm data denoting a rod-like micelle shape^35^. In fact, the slope becomes steeper as the additive concentration increases demonstrating that the micelle topology becomes more elongated. For higher q values the curve slope increases once again owing to the sharp interface between the micelles and the surrounding solvent^35^.

A common manipulation of the neutron-scattering data is to plot the quantity *I(q)·q* over *q*, referred to as Holtzer plot and depicted in Fig. 1b, which offers additional information on the structural properties of the micelles forming in the stagnant solvent. Firstly, the curves exhibit a clear maximum point for all concentrations indicative of micelle elongation. The maximum location *q*_*max*_ can be utilised to derive the micelle gyration radius *R*_*g*_, according to the relation *u* = *q*_*max*_*R*_*g*_, with u = 1.41–1.78 depending on molecule chain dispersity^36^. As can be discerned, the location of the maximum shifts to lower values as concentration increases and hence the gyration radius increases to an indicative value of 12.11 nm for a concentration of 600 ppm from a respective value of 7.70 nm for 100 ppm (considering poly-disperse molecule chains). Therefore, it can be deduced that for relatively high concentrations the micelles become elongated and have the potential to behave in a flexible manner capable of impeding flow motion under dynamic flow conditions^37^.

Another distinctive feature of the Holtzer plot is a plateau forming for higher q values, compared to the characteristic maximum point, with their relative height-wise distance being indicative of the number of persistence lengths per macromolecular chain and, hence, of the micelle flexibility^36^. Nevertheless, as also illustrated by the magnified view provided as an inset in Fig. 1b, a plateau cannot be clearly discerned in the high signal-to-noise region of the plot (*q* < *0.1*) and therefore a stiff micelle morphology should be postulated.

In summary, the interpretation of the SANS data verifies that QAS additives form rod-like micelles in stagnant dodecane and, furthermore, that the micelles exhibit the morphological characteristics required for the formation of complex, ‘net-like’ aggregates under flow conditions. The flow behaviour of additised diesel fuel at conditions with relevance to fuel injection equipment is presented in the next section.

### Cavitation topology and dynamics

**Figure 2:**
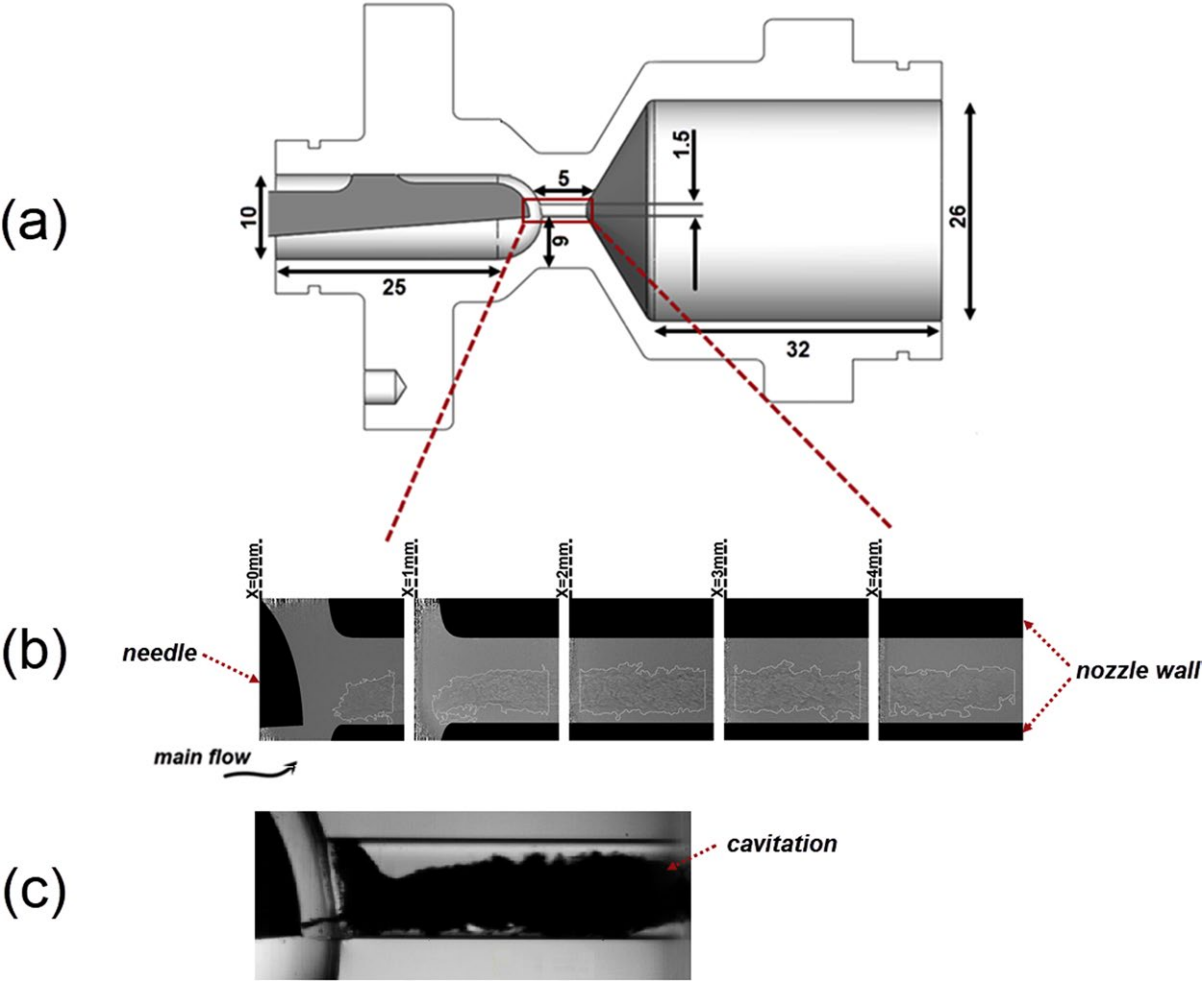
(**a**) Schematic and basic dimensions of the orifice internal geometry (all dimensions in mm). The material thickness was chosen equal to 6 mm, which ensures structural rigidity of the test piece, while it allows the acquisition of radiographs with adequate signal-to-noise ratio. The uncertainty in the orifice dimensions lies in the range 19 μm (curved sac) to less than 4 μm (orifice diameter). (**b**) Sample of irradiated regions along the orifice length. The beam diameter is 2.5 mm yet successive regions where irradiated at 1.0 mm intervals to allow for overlap at the locations of high noise. It must be noted that locations were irradiated in a ‘serial’ manner, i.e. from X = 0.0 mm to X = 4.0 mm. The white line denotes the cavity interface. (**c**) Indicative side-view raw image produced by high speed shadowgraphy illustrating the vapour content within the orifice (black cloud) for well-established cavitation (Re = 35500, CN = 3.0). It must be pointed out that the cavity structure appears opaque thus preventing the illustration of its topology in the channel core.

An axisymmetric carbon-fibre orifice has been employed in the X-ray flow-visualisation investigation with a geometrical constriction approximately at its mid length, as depicted in Fig. 2. A static, yet adjustable, metallic needle has been inserted upstream the constriction in order to replicate, in a simplified manner, the geometrical topology of a fuel injector. A more detailed discussion on the orifice design and manufacturing processes along with the rationale behind them can be found in a previous study of the authors^21^. Six test cases per fluid blend are presented in total, as summarized in Table 1, characterized by different cavitation numbers and Reynolds number of 35500. Indicative images of the cavitating flow as illustrated by high-speed shadowgraphy for a transparent (acrylic) orifice with identical nominal layout are also presented in Fig. 2. Besides, two needle-lift positions have been considered equal to 0.5 mm and 1.0 mm respectively, which have been verified to trigger different cavitation regimes, as will be discussed thoroughly in the paragraph below. Time resolved, side and top-view radiographs have been obtained through XPCI for the entirety of the orifice length; nevertheless, it is essential to emphasize that the orifice has been successively irradiated along five regions due to the collimation of the X-ray beam.

**Table 1:** Matrix of experimental test cases.

Case no.	Needle lift (mm)	Re	CN	P_inj_∙10^5^ (Pa)	P_back_∙10^5^ (Pa)	P_sat_ (Pa)
1	0.5	35500	3.0	36.6	9.3	21000
2			4.0	34.7	6.9	21100
3			7.7	31.8	3.8	20700
4	1.0	35500	3.0	30.0	7.4	21300
5			4.0	27.6	5.7	21000
6			6.0	26.4	3.9	20900

The exact same flow conditions and needle lift have been applied for the base and additised diesel blends examined.

**Figure 3:**
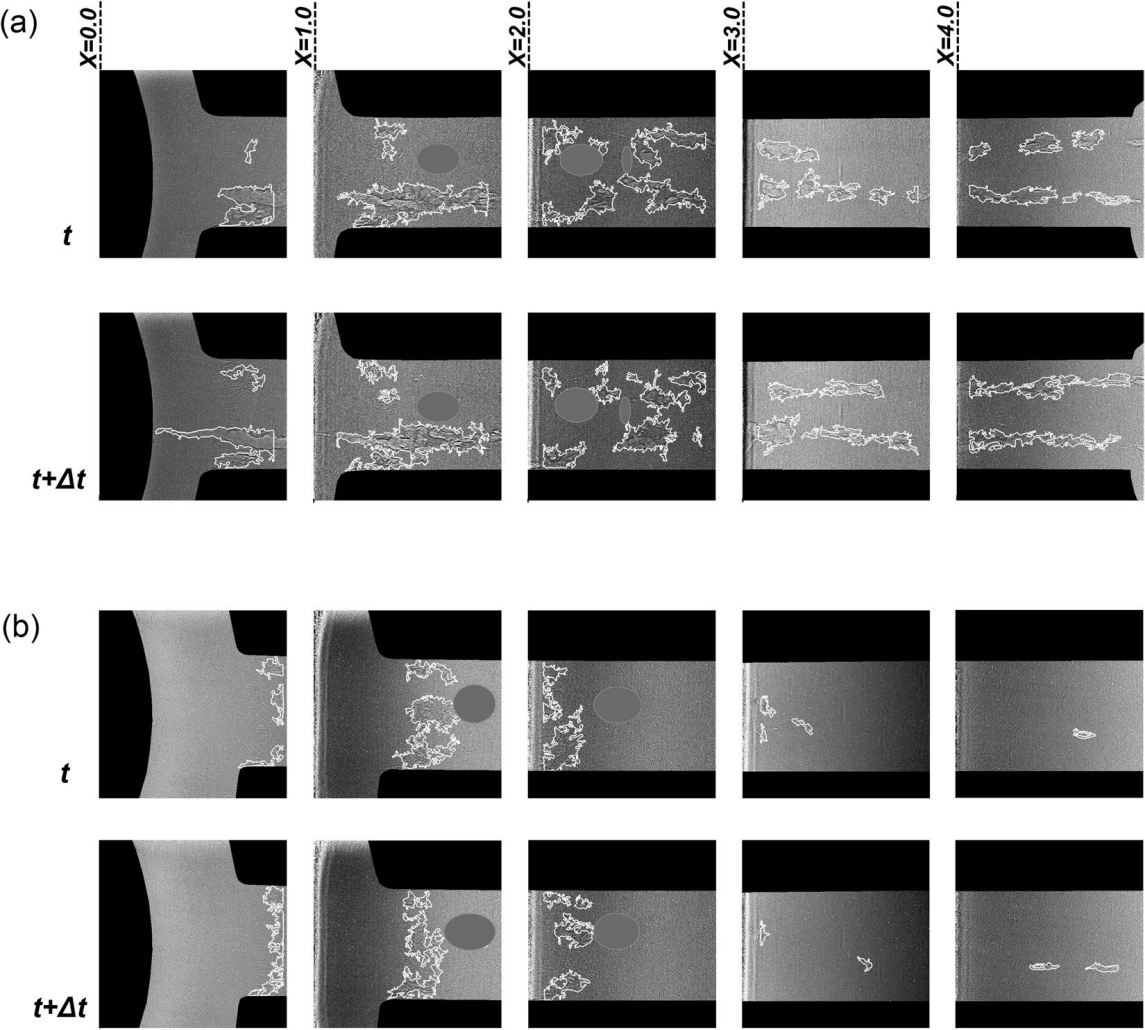
Top-view sequence of post-processed radiographs along the orifice length. The white line corresponds to the liquid-vapour interphase, while vertical white edges signify the extent of the irradiated region for which high signal-to-noise ratio could be obtained. Artefacts attributed to imperfections of the scintillator have been omitted in grey colour. (**a**) Needle lift equal to 0.5 mm for which string cavities arise in the orifice. (**b**) Needle lift equal to 1.0 mm giving rise to cloud cavitation. Both figures (**a**) and (**b**) present radiographs for CN = 3.0 and Re = 35500. The time interval *dt* is equal to 1.473 × 10^−5^ s.

Figure 3 presents post-processed radiographs illustrating the form and evolution of vaporous structures along the orifice length. The white line corresponds to the liquid vapour interface. It should also be noted that the grey spots evident in Fig. 3 (regions corresponding to *X* = *1.0 mm* and *X* = *2.0* *mm*) cover artefacts present in the radiographs caused by imperfections of the scintillator crystal that have arisen due to prolonged exposure to the high-flux X-ray beam. For a needle lift equal to 0.5 mm (Fig. 3a), elongated vaporous structures occupy the entire length, i.e. string cavitation constitutes the prevailing regime. A pair of cavities is clearly discernible, with the two structures interacting as the cavitation number increases. Moderate vapour generation is also observed attached to the wall in the vicinity of the nozzle entrance, especially at the side from which the main flow enters the orifice (lower side of the figures). Vapour formation in the wall region indicates that flow separation does occur downstream the geometrical constriction, nevertheless it is not of considerable magnitude and extent so as to give rise to a well-established cloud cavity. On the contrary, for needle lift equal to 1.0 mm, an attached cloud cavity establishes at the orifice entrance region and occupies the entire orifice cross-section. Cases characterised by higher cavitation numbers (not depicted in Fig. 3b for brevity) have verified that the cloud cavity expands further to the downstream and the cloud becomes unstable at the closure region with vortical structures being shed at high frequencies.

The distinct variation in the form of cavitation is attributed to the modification of the secondary flow field due to the extent of constriction realized at the region between the needle and its seat. In other words, the presence of coherent longitudinal and/or cross-flow vortices of significant intensity within the orifice influences the cavitation topology to a great extent and gives rise to the respective cavitation regimes. In fact, time-resolved XPCI data can be utilised to quantify the vorticity magnitude of the underlying whirling-flow motion^21^.

For a 0.5 mm needle lift, the emanation of the string cavity from the needle tip suggests that the underlying cause is a longitudinal vortex, as explained in^21^. On the other hand, for lift equal to 1.0 mm, cavitation is completely absent in the region upstream the orifice entrance and the cloud is attached to the nozzle wall. Hence, cavitation onset in this case is owed to the downstream flow separation, giving rise to a cross-flow vortex, a distinct feature of constricted flows^38^. Of course, in three-dimensional flow layouts, such as the one examined, a complex recirculating flow pattern is probable to emerge comprising both longitudinal and cross-flow vortices (flow separation). The relative recirculation magnitude is designating the cavitation topology in each case. The effect of confinement on a cavitating tip vortex has been examined by Boulon *et al*.^39^ who have also attributed the transition from string to cloud cavitation setting in at the tip of an ellipse-shaped head form to the underlying secondary-flow field. Besides, the interaction of coherent, large-scale cavitating vortices with small-scale vortices, i.e. turbulence, also has a considerable influence on the cavitation topology and dynamics, which has been verified to be more pronounced in the string-cavitation regime, as will be discussed in further detail in the Discussion section and has also been demonstrated by the authors in^21^.

**Figure 4:**
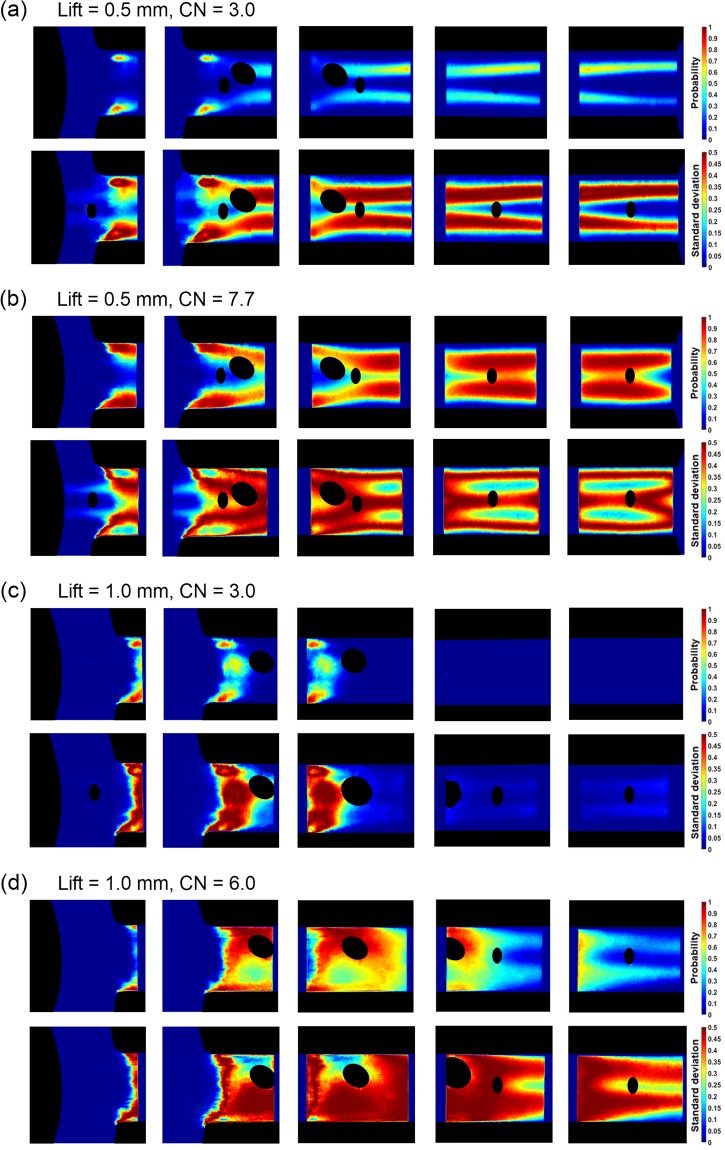
Mean and standard deviation images (top view) for the base blend produced by 16000 radiographs for each irradiated location (Re = 35500). Mean (top frame) and standard deviation (bottom frame) images for (**a**) lift = 0.5 mm-CN = 3.0, (**b**) lift = 0.5 mm-CN = 7.7, (**c**) lift = 1.0 mm-CN = 3.0 and (**d**) lift = 1.0 mm-CN = 6.0 (maximum CN achievable for the specific lift). The probability values of the legend refer to the vapour presence at each pixel of the plot, while the standard-deviation values denote the probability variation and are indicative of transient cavitating structures. The respective images for the additised blend exhibit similar trends and the same overall qualitative patterns regarding both mean probability and standard deviation. Artefacts attributed to imperfections of the scintillator have been omitted in black colour. Flow is from left to right.

Figure 4 presents contour plots of mean and standard deviation images produced by 16000 radiographs obtained for each location and test case examined. The black spots discernible in the frames of Fig. 4 correspond to regions, where probability and standard deviations values could not be calculated due to artefacts present in the raw radiographs, as also reported in the discussion referring to Fig. 3. For the low needle lift case (Fig. 4a) and moderate cavitation numbers (CN = 3.0), the flow is highly fluctuating as demonstrated by the low mean probability and high standard deviation values. On the contrary for the highest cavitation number obtained (CN = 7.7) the vortical cavities obtain relatively steady cores with considerable interfacial perturbations (high standard-deviation values), while a strong interaction is evident between the two structures. The respective plots for needle lift of 1.0 mm (Fig. 4b) clearly demonstrate a three-dimensional cloud cavity setting in at the orifice entrance. For CN = 3.0, the averaged and stand deviation images illustrate that the cloud occupies only the entrance region of the orifice, exhibits some topological fluctuations, yet no vapour structures of significance appear for *X* ≥ *2.5* *mm*. Nevertheless, a close look at the standard deviation image for the region in mention hints that a shedding sequence is on the verge of commencing, as very low, however non-zero values, are obtained for *X* > *3.0 mm*. For CN = 6.0 an extensive vapour cloud, with pronounced asymmetry in the cross flow direction occupies the largest part of the orifice. The distinct three-dimensional cloud form is also enhanced by fabrication imperfections, e.g. variations in the orifice entrance curvature. A strong shedding sequence of highly fluctuating structures, denoted by the high standard deviation values, is illustrated at locations close to the orifice outlet (*X* > *3.0* *mm*).

**Figure 5:**
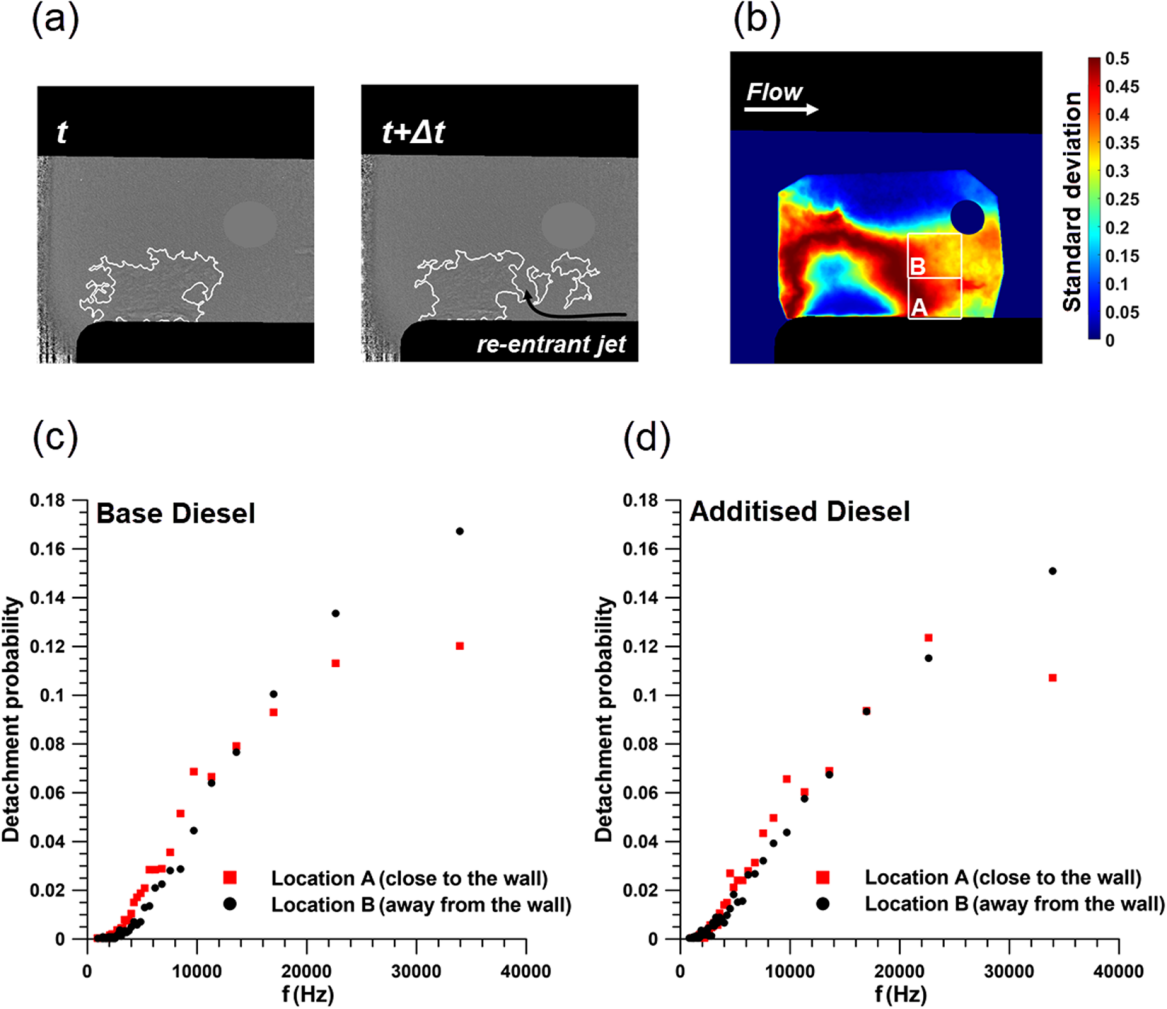
Cloud vortex shedding for lift equal to 1.0 mm, Re = 35500 and CN = 4.0. (**a**) Sequence of side-view radiographs (base diesel) illustrating the two shedding locations identified. (**b**) control windows for detachment detection superimposed on contour plot of standard deviation (base diesel). As can be seen, the windows are properly placed on regions of highly fluctuating cavitating features. Shedding frequency probability for (**c**) base and (**d**) additised diesel. The maximum experimental uncertainty for the frequency values is equal to 1.7% and is designated by the exposure time of the imaging technique.

The vortex-shedding sequence manifested for high CN is also clearly evident on the side-view radiographs depicted in Fig. 5a. In fact, two detachment locations (A and B) have been identified in reference to the vertical distance from the nozzle wall, as shown in Fig. 5b. The detachment frequency for the two locations has been calculated for the two fuel blends and is presented in Fig. 5c,d. A higher prevailing frequency, indicative of a more transient flow field is obtained at the location away from the nozzle wall (location B) in both cases. The most commonly encountered mechanism responsible for shedding is the, so called, re-entrant jet formed due to the adverse pressure gradient at the cavity closure region^40^. In other words, a secondary-flow motion with opposite direction compared to the main flow arises. Once a region of increased pressure is encountered, this secondary flow shifts upwards and severs a part of the cavitating cloud. It is interesting to notice that the prevailing frequency at location A for the additised fuel shifts to a lower value compared to the base diesel, indicating that the momentum of the re-entrant jet is decreased under the presence of additives, an assumption, which is in agreement with findings available in the literature^41–43^. With reference to location B, the shedding sequence retains its high frequency for both fuel blends. The additional perturbations inflicted by the longitudinal vortices present in the channel core seem to be contributing to the action of the re-entrant jet and hence the detachment is more frequent compared to location A. It is important to note that for the specific location, the prevailing shedding frequency probability (approximately equal to 25% and 18%, respectively for the base and additised blends, respectively) lies above the temporal resolution of the technique employed, thus demonstrating the transient nature of the flow.

**Figure 6:**
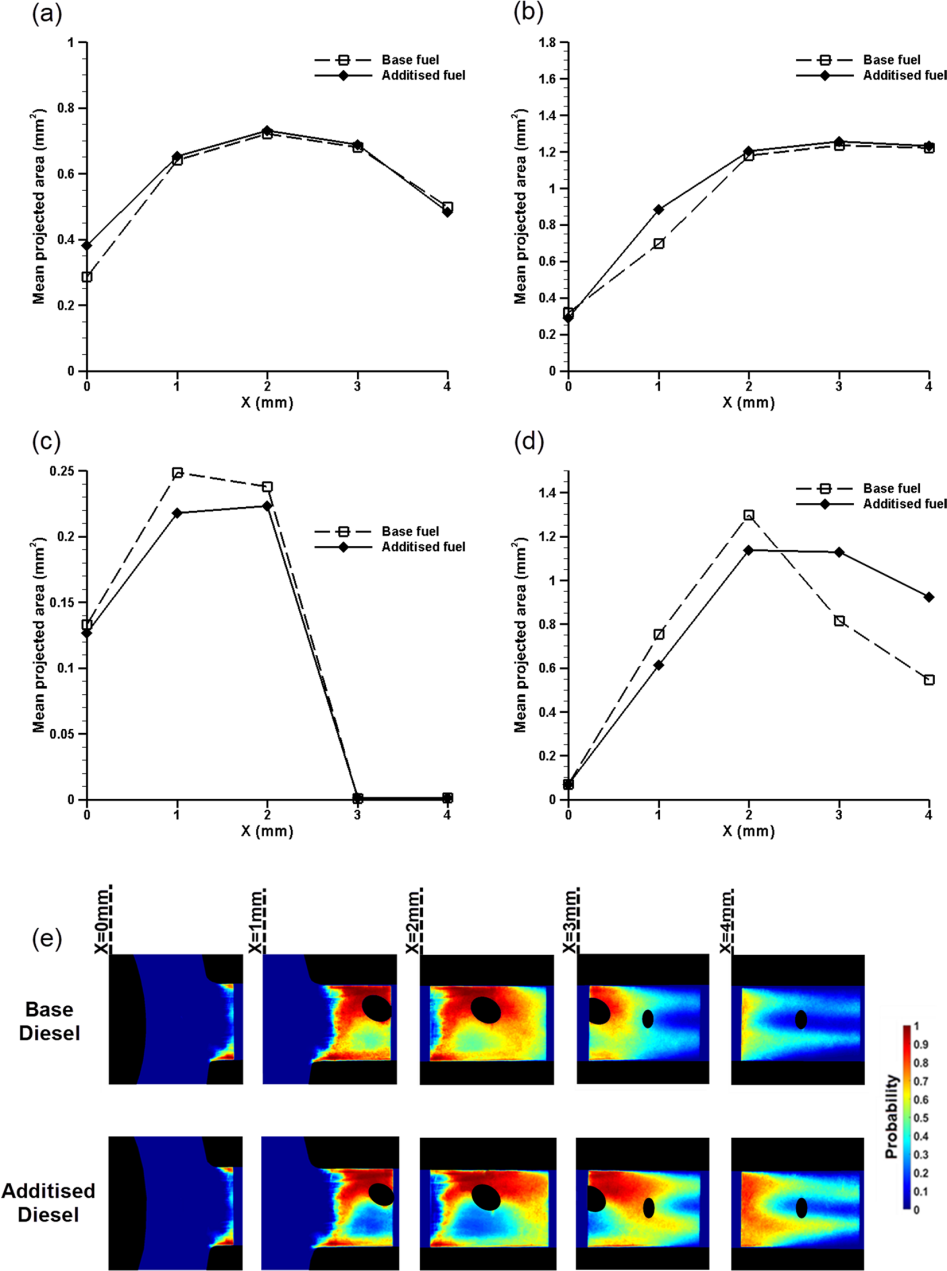
Mean surface projection of the cavity along the orifice length. Τhe values of the horizontal axis correspond to the regions irradiated (refer to Fig. 2): Needle lift of 0.5 mm: (**a**) CN = 4.0 and (**b**) CN = 7.7. Needle lift of 1.0 mm: (**c**) CN = 3.0 and (**d**) CN = 6.0. (**e**) Contour plots (top-view) of the mean vapour-presence probability for the base (top frame) and additised (bottom frame) diesel blends (Needle lift of 1.0 mm, Re = 35500, CN = 6.0). The plot corresponding to the base diesel is also depicted in Fig. 4d and has been added to facilitate the comparison between the blends. The maximum experimental uncertainty associated with the surface projection values lies in range 0.6–1.4% and it is designated by the spatial resolution of the imaging technique.

The major objective of the work presented is to verify the effect of QAS additives on cavitation formation. Along this direction, Fig. 6 presents the vapour mean projected surface for the two cavitation regimes that emerge depending on the needle lift. It is essential to remind at this point that contrast fluctuations in XPCI occur due to strong index gradients and, hence, are representative of the volume vapour content. In essence, the two-dimensional cavitation projections are not vulnerable to excessive irradiation scattering, as would be the case for optical methods, which would render the measurements inaccurate. Figure 6a,b refer to the 0.5 mm needle-lift case for moderate to high cavitation numbers and Reynolds number equal to 35500. Relatively stable string cavities have established for these conditions as demonstrated by Fig. 4b. With regard to 0.5 mm lift, the cavity projected area is higher for the additised diesel, 33% and 27% maximum for CN = 4.0 (Fig. 6a) and CN = 7.7 (Fig. 6b), respectively. In essence, the presence of additives enhances string cavitation. The maximum variation is exhibited in the orifice entrance region (X ≤ 1.0 mm), where the longitudinal vortices giving rise to cavitation obtain their maximum magnitude.

On the contrary, for needle lift equal to 1.0, the effect of additives becomes ambiguous, as depicted in Fig. 6c,d. In the case of CN = 3.0 (Fig. 6c), the influence of QAS additives is reversed compared to 0.5 mm, as their presence reduces the cloud cavity projected surface in comparison to base diesel by approximately 12.5%. The specific case (CN = 3.0) has been selected, as it corresponds to well-established cloud cavitation, however without shedding at its trailing edge. Notwithstanding, the plot for CN = 6.0 (Fig. 6d) exhibits an equivocal behaviour; QAS additives tend to reduce by approximately 18.5% the overall cavity surface up to X = 3.0, while, on the contrary, further downstream and approaching the orifice outlet, cavitation is significantly increased, by about 69%, compared to the base blend. A direct comparison between the vapour-presence probability within the orifice for the two blends and for flow conditions characterized by Re = 35500 and CN = 6.0 is offered by Fig. 4e. It is clearly illustrated that, although, the presence of additives has no influence on the qualitative morphology of cavitation, in the sense that the same cavitation regimes set in with regard to both blends, the vapour-content probability for the additised diesel, in general, obtains lower values in the cloud region (*X* ≤ *3.0* *mm*). On the contrary, higher probability values compared to the reference blend appear in the trailing edge from which cavitating vortices are shed (*3.0* *mm* ≤ *X* ≤ *5.0* *mm*). The same observations have been found to be consistent for all the cases examined referring to both string and cloud cavitation, namely the presence of additives does not alter the cavitation form yet it modifies the vapour overall extent from a quantitative point of view.

The available data in combination with the SANS measurements suffice to draw general conclusions on the effect of additives concentration on the cavitation topology, although a direct comparison between blends of lower concentration (300 and 100 ppm) and base Diesel has not been performed. Additised blends with different concentrations are therefore expected to exhibit the same cavitation-form regimes as the base blend, while the quantitate differences in the overall vapour extent within the orifice is expected to increase with increasing additive concentration, as the viscoelastic micelles become of more considerable size. A full interpretation of the flow behaviour of the QAS-additised fuel must be sought on the interaction of the viscoelastic micelles with the arising vortical structures, as will be discussed in detail in the following paragraph.

## Discussion

Cloud cavitation manifestation is, in essence, always associated with the presence of a separated shear layer. Hence, for the nozzle flow investigated in this study the cavitation topology and dynamics are influenced by the secondary flow pattern, the level of turbulence in the orifice and, especially referring to the additised fuel blend, the interaction of QAS micelles with vortices at different scales. Single-phase turbulent viscoelastic flows have been extensively investigated in parallel and separated flows and some of the results established can be directly extrapolated to cavitating flows as well. Viscoelasticity has been proved to affect the turbulent boundary layer and therefore the flow shear.

**Figure 7:**
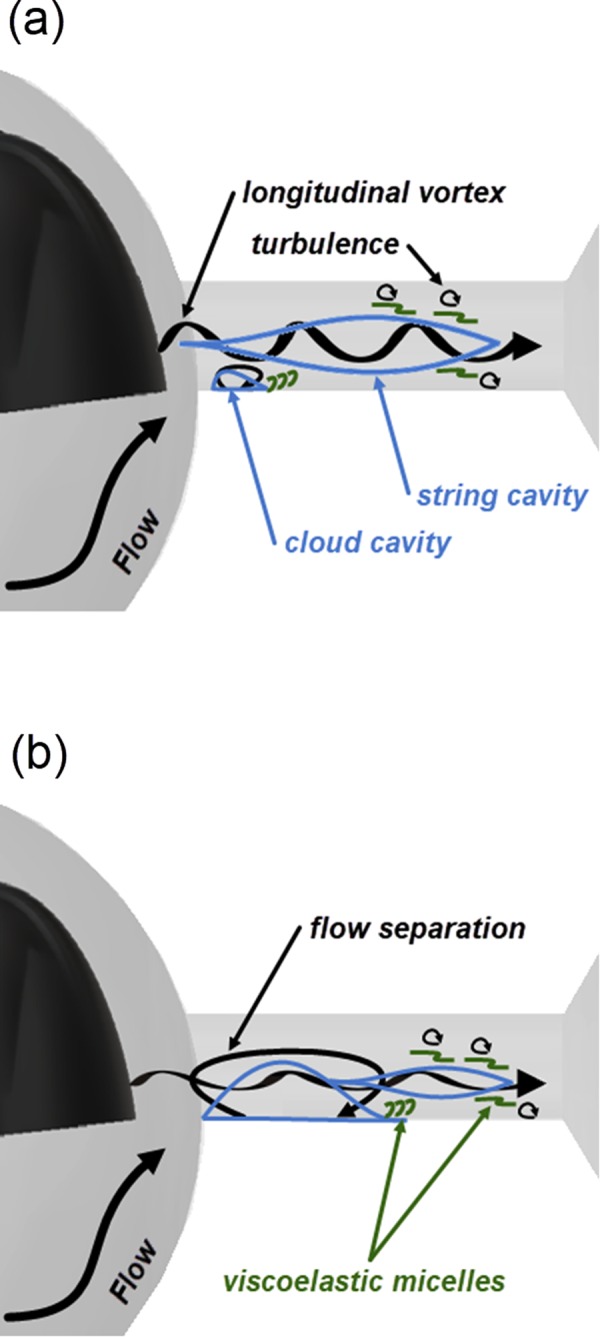
Schematic illustrating the secondary flow pattern, cavitation topology and postulated action of QAS micelles within the orifice for the different cavitation regimes identified. Parallel and vortical flow motion is represented by black arrows, cavitating structures by blue closed iso-lines and micelle structures by a network of curved green lines. Lift of (**a**) 0.5 mm and (**b**) 1.0 mm.

As depicted in the schematic representation of Fig. 7a, the underlying cause for the attached cloud cavity emerging for needle lift equal to 1.0 mm, is postulated to be the three-dimensional separation downstream the constriction, a distinct feature of abruptly-contracted flows. The vorticity magnitude of the span-wise vortex formed in the region is vital for the morphology of the cloud cavity. It has been verified through DNS that polymers exhibit higher deformation in the near-wall region compared to the core flow and physically oppose the vortical motion and transform vortex kinetic energy to polymer elastic energy^44,45^. Damping of spanwise vortices downstream of a flow-contracting layout has also been experimentally verified in^26^ through PIV measurements.

Longitudinal vortices also commonly arise in three-dimensional constricted flows, e.g. forward facing steps, owing to boundary layer perturbations upstream the constriction^46^. Such vortices are entrained by the main flow, persist downstream and are eventually damped through the interaction with small-scale vortices, i.e. flow turbulence. As has been emphasized in the literature, under the presence of viscoelastic additives the turbulent cascade to the dissipative scale is terminated prematurely, and scales below a certain limit, designated by the elasticity of the micelles interact with the polymer and not with vortices of larger scales^47^. Therefore, viscous damping of the vortices is delayed allowing the string cavities to live longer, as indicated in Fig. 7b. Tsukahara *et al*.^26^ have also observed enhanced intensity and longer lifetimes of the longitudinal vortices downstream a flow constriction for viscoelastic fluid flow compared to water.

**Figure 8:**
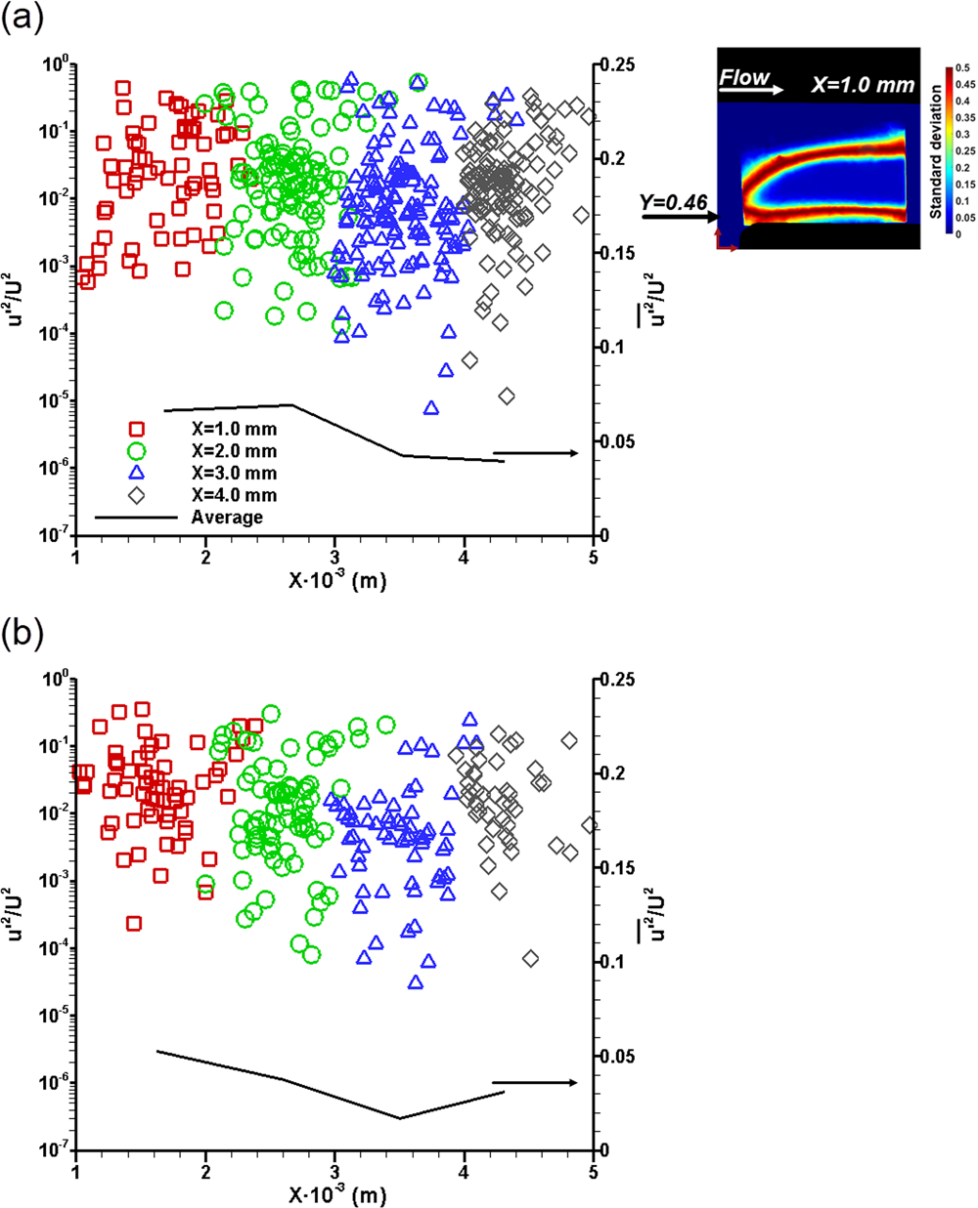
Level of turbulence within the orifice for Re = 35500, CN = 4.0 and needle lift of 0.5 mm: (**a**) Base and (**b**) additised diesel. As shown by the inset of (**a**) the point velocity measurements were obtained from the side view radiographs at a location corresponding to Y ≈ 0.46 ± 0.04 mm for which cavitation presents transient features corresponding to high standard deviation values. The black line corresponds to the averaged quantity per irradiation location. The experimental uncertainty associated with *u*^*’2*^*/U*^*2*^ lies in the range 5.5–5.9%, while the uncertainty in the axial-location values is equal to 2.5%.

In order to further support the argument that the enhanced string cavitation in the presence of QAS additives is owed to the decreased interaction of the prevailing longitudinal vortices with vortical structures of smaller scales, an estimate of the level of turbulence along the orifice length is given. Figure 8 depicts the axial-velocity fluctuation constituent *u’* in non-dimensional form for the base and additised blends. The local velocity field was measured at the nozzle regions where the cavity topology exhibits high standard deviation with a technique bearing resemblance to PTV. Further information on the velocimetry method are given in the next section and also in^21^. In brief, a considerable number of planar velocity values were measured at cavity regions with distinct interfacial features or fully separated structures with sizes below 700 μm. The deviations of the measured axial velocity constituent from the average flow velocity *U* calculated by the flow rate imposed during the experiment constitute the velocity fluctuation *u’* presented in Fig. 8.

A comparison between Fig. 8a,b reveals that the degree of velocity fluctuations are suppressed throughout the orifice length under the presence of QAS additives in the diesel fuel for CN = 4.0, a value for which well-established strings prevail in the entire orifice length. The averaged quantity denoted as a black line in both graphs suggests a decreasing trend for the additised fuel up to the last part closer to the outlet. The slightly increased fluctuations observed in that region can be attributed to the cavity partial collapse in the vicinity of the orifice outlet, which has a more pronounced effect on the level of turbulence compared to the base blend.

To summarize the main findings of the present study, SANS and XPCI have been demonstrated as a suitable set of techniques to thoroughly characterise the rheological and flow behaviour of diesel fuel with suspended QAS additives in a turbulent wall confined flow with resemblance to fuel-injection conditions. SANS has revealed the presence of rod-like micelles in stagnant diesel surrogate, which become elongated and retain a ‘stiff’ morphology as the additive concentration increases. Time resolved XPCI of the flow field within an enlarged injector replica has illustrated the formation of string or cloud cavitation depending on the degree of flow constriction (needle lift). A strong vortex shedding sequence has been detected for a needle lift of 1.0 mm and moderate to high cavitation numbers. The presence of QAS additives has been verified to enhance string yet suppress cloud cavitation. The fundamental physical mechanism for this distinct flow behaviour is associated with the interaction of viscoelastic micelles with vortical flow at different scales and can be extrapolated from single-phase flows for which there is experimental evidence in the literature. The damping of coherent stream-wise vortices is delayed due to turbulence suppression, i.e. suppression of small-scale vortices, induced by the micelles; hence string cavitation is enhanced. On the contrary, complex, ‘net-like’ micelle aggregates are postulated to form in the separated boundary layer region, modify the local shear rate and induce cavitation suppression in the region. Apart from the influence on cavitation formation, the addition of QAS additives on the base fuel has been found to reduce the level of turbulence throughout the orifice.

The dataset obtained in the present study constitutes experimental evidence of the effect of QAS viscoelastic additives on the development and collapse of different forms of cavitation. To the authors’ knowledge temporally-resolved information with reference to non-Newtonian two-phase flows is widely missing from the relevant literature. The obtained results verify that conclusions drawn for single-phase flows regarding the influence of viscoelasticity on coherent vortical motion are also applicable to two-phase flows. In fact, the physical mechanism leading to different behaviour of string and cloud cavitation compared to Newtonian flow has been revealed to be primarily designated by secondary-flow processes. Overall, the observations are consistent with the enhanced fuel-delivery efficiency of commercial fuel injectors, which has been observed and reported in^48^. Effective injection process results in power gain of the entire IC engine with profound impact in the fields of land and marine transportation, as well as energy production.

## Methods

### Small Angle Neutron Scattering

The SANS2D neutron source employed for the neutron scattering measurements produces an 8.0 mm in diameter neutron beam with a typical time-averaged flux of the order of 10^6^ cm^−2^ s^−1^. The scattering vector obtained by the specific instrument lies in the range 0.005–0.491 Å^−1^. Two 3He-CF4 filled ORDELA^49^ detectors with active area of 96.5 cm × 96.5 cm and spatial resolution of 5 mm were utilised for the scattered neutron count. So-called “banjo” shaped sample holders were used for the stagnant solvent/additive blend having a thickness of 5.0 mm along the path length. The significant duration of the measurements along with the high neutron flux have been found to increase the signal-to-noise ratio of the obtained data. The beam scattering induced by the solvent and sample cell have also been separately measured and used to correct the raw measurements. Normalisation of the data into absolute units has been achieved using a partially-deuterated known polymer. The sample temperature was maintained at 25 ± 0.5 °C and monitored through two type K thermocouples, located in the sample-changer environment and inserted inside the scattering cell, respectively. Different samples were allowed to reach equilibrium at the specified temperature for at least 30 minutes prior to testing. Deuterated dodecane (98 atom % 2D) was considered as a representative diesel-fuel surrogate. Medium deuteration is necessary to enhance the contrast variation between the forming micelles and the solvent, as it increases the scattering length density compared to hydrogen. The samples to be tested were diluted with the solvent until the desired additive concentration was reached. The blend was subsequently mechanically stirred and left to rest for 24 hours.

### X-ray Phase Contrast Imaging

**Figure 9:**
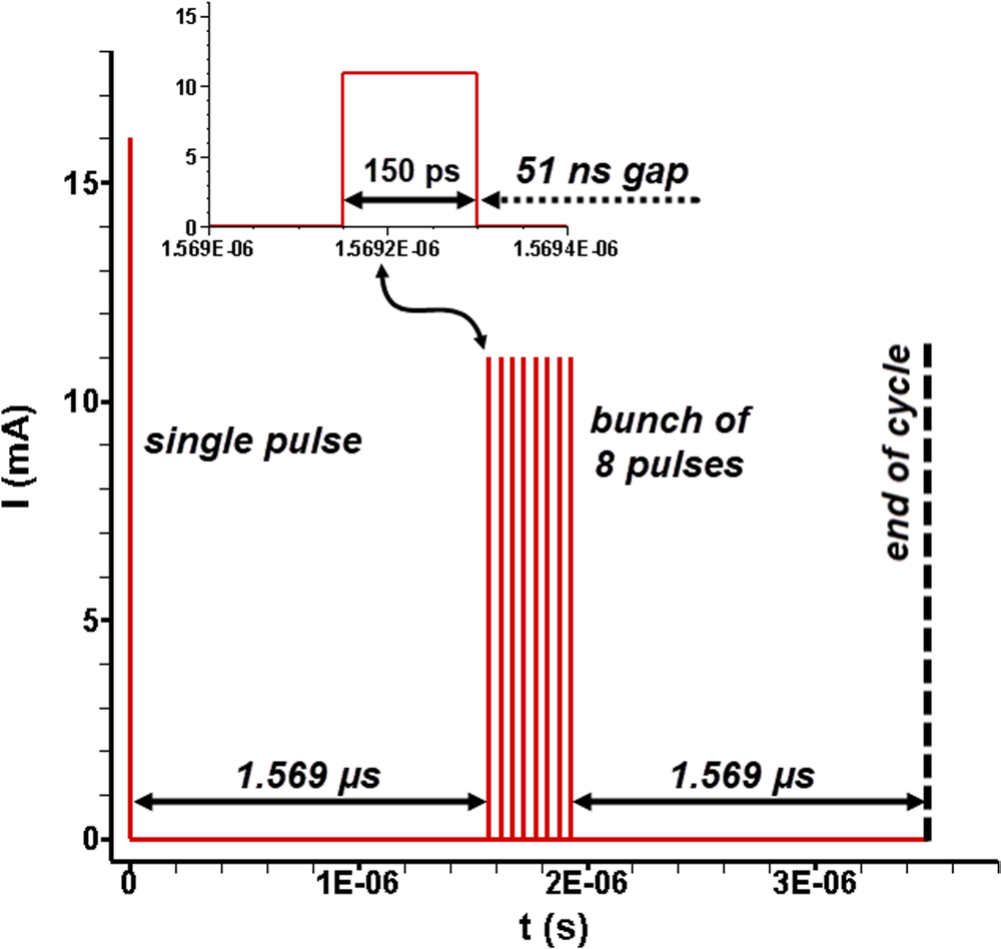
Beam pulsation mode. A high-current (16 mA) pulse is followed by a bunch of eight pulses each one carrying 11 mA. A gap of 51 ns intervenes between successive pulses of the bunch. Six out of the eight pulses comprising the bunch were employed in the present investigation.

XPCI produces images with contrast fluctuations due the X-ray wave phase shift incurred by the beam scattering, as it interacts with matter. Nevertheless, the beam scattering induced by the liquid bulk is orders of magnitude lower compared to the respective for visible light^22^. In other words, fine features of the two-phase flow, usually obscured by excessive scattering in conventional optical imaging are highlighted through XPCI. A white X-ray beam with energy of 12 keV available at beamline 7-ID of the Advanced Photon Source was used for the experiments. The beam is generated at the synchrotron storage ring and guided by an undulator to the beamline. Its pulsation mode is shown in Fig. 9 and comprises nine pulses per cycle. A scintillator crystal (LuAG:Ce) placed at the opposite side of the irradiated sample converts X-ray radiation to visible light (420 nm), which is subsequently captured by a metal–oxide–semiconductor (CMOS) type high-speed camera (Photron SA-Z). The beam pulsation mode made possible a shutter exposure time of 347 ns to be achieved, considerably lower compared to optical imaging^50^. Therefore, image sharpness is increased and fine features of the cavity interface can be accurately resolved. Top and side-view radiographs were obtained at 67890 frames per second with a field of view of 2.56 mm × 2.56 mm discretized by 512 × 512 pixels, thus, resulting to a spatial resolution of 5 μm/pixel. It must be reminded that due to the beam collimation the overall orifice length was irradiated in a successive manner, i.e. with a time interval between irradiation events required for the translational base on which the test sample was accommodated to reach the next region of interest.

### Image Post Processing

1000 twelve-bit greyscale images were obtained per irradiation event, so as to avoid overheating the scintillator. Nevertheless, 16000 images were taken in total for each location irradiated, in order to verify the periodic nature of the flow. All averaged quantities were calculated using the full set of 16000 images. The post-processing procedure of the raw images comprised a series of techniques with the objective to identify the interface between the emerging cavitating structures and the surrounding liquid. In a first step, the raw radiographs were divided by stagnant liquid (background) images to enhance contrast. The phase congruency method^51^ was subsequently applied to highlight salient image features. Application of a mean filter and proper thresholding produced binarised images making the interface detection feasible using Canny’s algorithm. A more detailed discussion on the post processing techniques employed to visualise the vapour interphase is provided as Supplementary Material, while the different post-processing steps are also reported in^21^.

Τhe identified vaporous structures enabled the estimation of the planar velocity field utilising a method bearing resemblance to Particle Tracking Velocimetry (PTV) according to which the motion of inherent flow features, such as bubble clouds or cavities of distinct shape, is tracked over successive time frames. The planar displacement of the mass centre of identified features in essence corresponds to the local flow velocity. PTV has been primarily realised until now in bubbly flows without phase change to derive the local velocity field and level of turbulence^32,33,52,53^. It must be pointed out that conventional Particle Image Velocimetry (PIV) has been proven problematic for velocity measurements in the vicinity of vapour regions with reference to cavitating flows, as the addition of tracer particles is possible to affect the cavitation onset^54^.

Taking into account the highly transient nature of cavitation, several precautions were taken during the post-processing of the radiographs to prevent the erroneous identification of newly-emerging vaporous structures as pre-existing cavities that have been convected by the main flow. Strict conditions were imposed with reference to the tracked vaporous structures size and location to ensure that the filtering process pinpoints invariant over time cavities and that no bias is introduced in the presented results. More specifically, only small-scale structures, with maximum size up to 700 μm, were tracked. The mean deviation in the projected area over consecutive frames of all tracked structures was calculated to be of the order of 8%. Besides, if successive frames exhibited different numbers of vaporous structures, i.e. a structure had emerged or collapsed, or structures were located in low signal-to-noise regions of the interrogation window, then such frames were omitted from further analysis. It is essential to point out that the imposed filtering conditions resulted in fewer than 1% of the 16 000 images obtained for each irradiated location being eligible for the velocimetry measurements. Nevertheless, the approximately 160 velocity vectors obtained for each 1.0 mm of orifice length are deemed as sufficient for the estimation of the local velocity field, judging by other PTV/PIV investigations of bubbly flows available in the literature^32,33^. A thorough outline of all the steps of the post-processing methodology employed to extract physical information regarding the vapour extent and the velocity field from the raw radiographs is also provided in^21^.

### Hydraulic loop and instrumentation

The hydraulic flow loop employed in the experimental investigation is thoroughly described in^50^ and was operated under steady-state flow rate conditions for the needs of the reported experiments. The prototype injector-replica orifice employed was manufactured from a carbon-fibre composite material (Torayca TF00S), which is capable of withstanding pressures of the order of 150 bars, while it causes low X-ray radiation attenuation compared to metals, thus enabling the acquisition of radiographs with high signal-to-noise ratio. A steel needle with a hemispherical tip was inserted into the sac region upstream the injector hole to replicate, in a simplified manner, the geometrical layout of a real-size fuel injector. The prevailing flow conditions within the injector orifice were characterised with the use of the Reynolds and cavitation numbers defined as follows:

1 $$Re=\frac{{u}_{ave}\cdot D}{{\nu }_{fuel}}=\frac{4\dot{V}}{(\pi D)\cdot {\nu }_{fuel}}$$

2 $$CN=\frac{{p}_{inj}-{p}_{o}}{{p}_{o}-{p}_{sat}}$$

Referring to the Reynolds number definition, *u*_*ave*_ is the flow average velocity within the orifice, *D*(=1.5 mm) is the orifice internal diameter, $\dot{V}$ is the imposed volumetric flow rate and *ν*_*fuel*_ is diesel kinematic viscosity^17,21,50^. The values of the injection *p*_*inj*_, back (outlet) *p*_*back*_ and saturation pressure *p*_*sat*_ are required to define the cavitation number. Diesel thermo-physical properties were obtained from the database available in^55^. It must be emphasised that the fuel macroscopic properties are not affected by the presence of QAS additives and therefore matching of the non-dimensional numbers for the two blends examined offers a straightforward comparison.

Fuel temperature was monitored through type-K thermocouples inserted at the fuel tank and the inlet/outlet manifolds and maintained at a temperature of 40.0 ± 1.0 °C through a water-cooled heat exchanger and a PID controller. Volumetric flow rate was regulated by a three-way valve located at the outlet of the feed pump and monitored through an axial-turbine flow meter. The back pressure was regulated by a valve located at the outflow manifold downstream the injector orifice outlet, while the upstream pressure adjusted to a value satisfying continuity for a specified flow rate. Pressure transducers inserted in taps located at the inlet and outlet manifolds were used to record the injection and back pressures. A typical error propagation analysis, as presented by Moffat^56^, has been followed to estimate the uncertainty associated with the relevant derived quantities presented in the results section. The Reynolds and cavitation numbers are associated with uncertainties equal to 3.1% and 5.3%, respectively.

## Electronic supplementary material

Supplementary Material

## Data Availability

The datasets generated and analysed during the current study are available from the corresponding author on reasonable request.
