# Supplementary material for: Illustrating the effect of viscoelastic additives on cavitation and turbulence with X-ray imaging
Source: Sci Rep. 2018 Oct 8;8:14968. doi: 10.1038/s41598-018-32996-w (PMC6175919; doi:10.1038/s41598-018-32996-w)
Supplement: Supplementary file 1 — Supplementary Material [file 41598_2018_32996_MOESM1_ESM.pdf]

Illustrating the effect of viscoelastic additives on cavitation and turbulence with X-ray imaging

I.K. Karathanassis[1,*], K. Trickett[2], P. Koukouvinis[1], J. Wang[3], R. Barbour[2], M. Gavaises[1]

[1]School of Mathematics, Computer Science and Engineering, City, University of London, EC1V 0HB, London, UK
[2]Lubrizol Limited, Hazelwood, DE56 4AN, Derby, UK
[3]Advanced Photon Source, Argonne National Laboratory, Lemont, IL 60439, USA
[*] Corresponding author

Contact information:
ioannis.karathanassis@city.ac.uk
kieran.trickett@lubrizol.com
foivos.koukouvinis.1@city.ac.uk
wangj@aps.anl.gov
robert.barbour@lubrizol.com
m.gavaises@city.ac.uk

Post processing of the Phase Contrast Radiographs

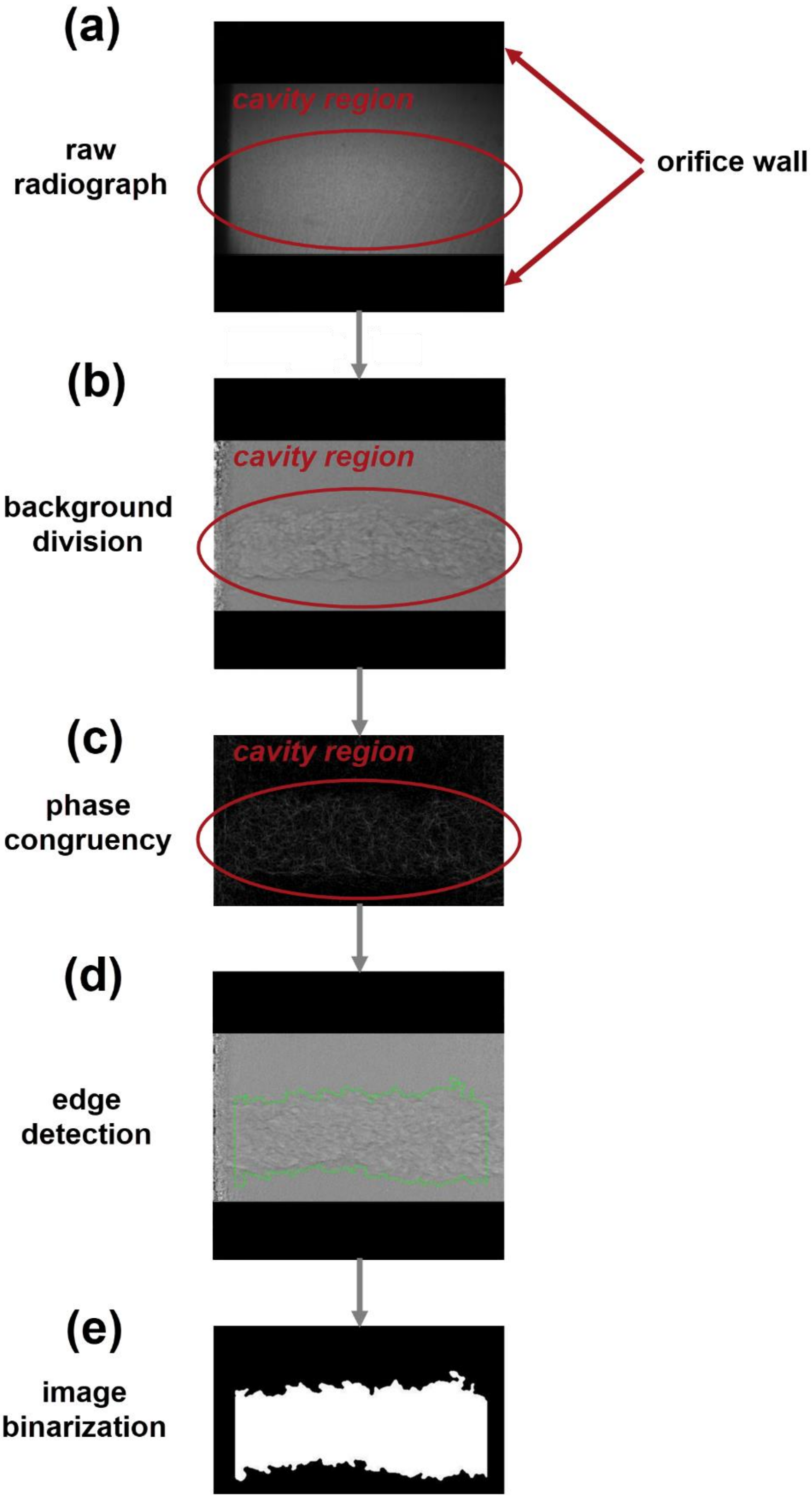

Figure S1. Post-processing methodology employed to derive the cavity interface from the raw phase-contrast images: **(a)** raw x-ray image, **(b)** image with enhanced contrast after division with background, **(c)** image with salient features highlighted (in white) after application of the phase congruency method, **(d)** image where the vapour cavity interface is detected (highlighted in green) using Canny's algorithm and **(e)** binarized image. The set of 16000 binarized images per characteristic location are subsequently used for the calculation of the vapour-presence probability and standard deviation depicted in Fig. 4.

The extraction of physical information regarding the vapour extent within the orifice from the raw XPCI data obtained required a series of post-processing techniques to be utilised, in order to enhance the image contrast and to highlight and detect the vapour/liquid interphase. The post-processing methodology was realised in an in-house Matlab code, also incorporating built-in functions of the Image Processing Toolbox and the Phase Congruency open-source algorithm (available at http://www.peterkovesi.com/matlabfns/). The methodology comprises the following steps, as also depicted in Fig. S1:

i) The contrast fluctuations in the raw radiographs are rather weak, as depicted in Fig. S1a and, hence, the image contrast has to be enhanced prior to the application of the edge detection algorithms.

ii) Division of each radiograph by a mean background image obtained at static flow conditions prior to each case examined results to the image depicted in Fig. S1b, where the contrast in the cavity region is significantly enhanced.

iii) The phase congruency method is subsequently applied to recognise the salient features of each image (Fig. S1c). The mathematical formulation of the method is described in detail in [51]. The primary advantage of the method over conventional, brightness-gradient based methods is its insensitivity in changes of image brightness or contrast. This is due to the fact that the obtained signal is expanded in a Fourier series and prevailing image features correspond to points of maximum phase congruency in the frequency (Fourier) domain. On the contrary, conventional edge-detection algorithms analyse images in the spatial domain and are, therefore, more susceptible to image illumination variation, as it affects the steepness of the pixel brightness gradient.

iv) Subsequently, a median filter is applied to remove noise from the phase-congruency image, without blurring the cavity interphase, which has been highlighted by the method. Canny's algorithm, searching for local maxima of the pixel-brightness gradient, is employed to detect the edges of the identified cavity structure, as depicted in Fig. S1d.

v) Finally, the grayscale image is converted to binary, Fig. S1e, by imposing a pixel brightness threshold, above and below of which all brightness values are substituted by 1 (white) and 0 (black), respectively.
